# Modulated theta band frequency with binaural beat stimulation correlates with improved cognitive scores in Alzheimer’s patients

**DOI:** 10.3389/fnagi.2025.1543282

**Published:** 2025-03-03

**Authors:** Muhammad Danish Mujib, Ahmad Zahid Rao, Muhammad Fahim Ul Haque, Ahmad O. Alokaily, Syeda Sehar Hussain, Ahmed A. Aldohbayb, Saad Ahmed Qazi, Muhammad Abul Hasan

**Affiliations:** ^1^Department of Biomedical Engineering, NED University of Engineering & Technology, Karachi, Pakistan; ^2^Department of Physical Medicine and Rehabilitation, McGovern Medical School, University of Texas Health Science Center at Houston, Houston, TX, United States; ^3^Department of Telecommunication Engineering, NED University of Engineering & Technology, Karachi, Pakistan; ^4^Department of Biomedical Technology, College of Applied Medical Sciences, King Saud University, Riyadh, Saudi Arabia; ^5^King Salman Center for Disability Research, Riyadh, Saudi Arabia; ^6^Department of Electrical Engineering, NED University of Engineering & Technology, Karachi, Pakistan; ^7^Neurocomputation Lab, National Center of Artificial Intelligence, NED University of Engineering & Technology, Karachi, Pakistan

**Keywords:** Alzheimer’s disease, binaural beat, cognitive score, correlation, EEG

## Abstract

**Introduction:**

Alzheimer’s disease (AD) affects 50 million individuals worldwide, a number projected to triple by 2050. Due to discomfort through electrical and magnetic neuromodulation technologies, this is the first study to propose the potential of auditory binaural beat (BB) stimulation at an alpha frequency (10 Hz) for enhancing cognitive and neurological outcomes in AD patients.

**Methods:**

Twenty-five patients were divided into the experimental-Group (*n* = 15) and control-Group (*n* = 10). Psychometric and neurological assessments were conducted Pre-Treatment (Day 1) and Post-Treatment (Day 14) following consecutive days of binaural beats (BB) or auditory tone stimulation administered from Day 2 to Day 13.

**Results:**

A two-way ANOVA revealed a significant main effect of group (*F* = 6.087, *p* = 0.016) and session (*F* = 3.859, *p* = 0.024) on MMSE scores, with the experimental group showing significant improvement in MMSE scores (*t* = 7.33, *p* = 0.00000012) compared to the control group (*p* = 0.2306). Paired *t*-tests revealed a significant reduction in depression scores (DASS-21, *t* = 1.701, *p* = 0.0253) in the experimental group, while no significant improvements were noted in the control group. EEG recordings revealed significant changes in α-band, β-band, and γ-band power (*p* < 0.05). Moreover, The correlation between EEG bands and MMSE subparts showed that increased θ-band power in the experimental group was positively correlated (*p* < 0.05) with the frontal region during language tasks and in the frontal and central regions during registration and orientation tasks, indicating potential neurocognitive benefits.

**Discussion:**

The results of this research imply that BB stimulation has untapped potential as a non-invasive therapy for patients with AD, hence there is the need for further studies to manage the dementia epidemic.

## 1 Introduction

Alzheimer’s disease (AD) is a neurological disorder that progressively impairs cognition, attention span, and memory and hinders the ability to perform everyday activities (Okabe et al., 2020; Ott et al., 1996; Parasuraman and Haxby, 1993). Behavioral and neurological symptoms of AD are associated with aging, as it is more prevalent in the elderly (Galasko et al., 1990; Turner et al., 2012). This condition progresses through three stages starting with memory loss in the early phase and learning difficulties in the intermediate stage and ultimately leading to complete debilitation in the severe stage (Agrawal et al., 2020; Galasko et al., 2005). According to the World Health Organization (WHO), around 50 million individuals worldwide are currently affected by AD and related dementia issues and this number is expected to triple by 2050 (World Health Organization, 2021).

Dementia in AD patients is a leading cause of dependency on professional caregivers for performing everyday activities (Bell et al., 2001; Galasko et al., 2005; González-Salvador et al., 1999). This dependency and inability to execute tasks on their own may have negative psychological effects on AD patients in the long term and thus lead to serious mental health issues such as anxiety, stress, and depression (Botto et al., 2022; Gallagher et al., 2011). According to previous studies, the Depression Anxiety Stress Scale (DASS-21) serves as a neuropsychological assessment tool to screen AD patients for signs of depression, stress, and anxiety (Ali et al., 2022). Effective assessment techniques for diagnosing the severity of AD include the Mini-Mental State Examination (MMSE) (Arevalo-Rodriguez et al., 2015), the Clinical Dementia Rating (CDR) (Morris et al., 2001), the Sum of Boxes (CDR-SB) (Williams et al., 2013), and the CDR orientation score (Kim et al., 2017). MMSE assesses a person’s cognitive abilities, specifically orientation, attention and recall, registration, calculation, language, and visual construction (MacKenzie et al., 1996; Sposito et al., 2015).

Aside from behavioral and neuropsychological assessments of AD patients, recent studies have also utilized neuroimaging techniques to investigate neurological changes. These include Positron Emission Tomography scan (Nordberg et al., 2010), Magnetic Resonance Imaging (Teipel et al., 2013), functional Magnetic Resonance Imaging (Machulda et al., 2003), Computed Tomography-scan (Cuttler et al., 2016), and electroencephalography (EEG) (Bennys et al., 2001). EEG provides frequency-specific changes in the cortex (García Domínguez et al., 2013; Soininen et al., 2020). Power spectrum density (PSD) is one of the features of EEG that may be used to compare the neurological changes that occur in AD (Liu et al., 2016; Wang et al., 2015). Increased θ-band power while decreasing α- and β-bands power is mainly reported specifically in the temporal and posterior/occipital brain regions (Azami et al., 2023; Babiloni et al., 2021; Bruña et al., 2023; Cassani et al., 2018; Horvath et al., 2018; López-Sanz et al., 2016; López-Sanz et al., 2019; Maestú and Fernández, 2020; Moretti et al., 2004; Musaeus et al., 2018; Roh et al., 2011).

Electrical and magnetic neuromodulation have been clinically proven as an effective means of treatment for the improvement of AD symptoms (Bentwich et al., 2011; Gangemi et al., 2021; Rajji, 2019; Scherder et al., 1995). Several studies have demonstrated, improved cognitive scores measured with MMSE following two to four weeks of neuromodulation with rTMS and tDCS (Im et al., 2019; Wei et al., 2022). In addition to potential risks to body tissues and feelings of discomfort, trained personnel are required to operate these neuromodulation techniques (Johnson, 2001; Nikolin et al., 2018; Russo et al., 2013). Therefore, it is impractical for family caregivers to provide these stimulations at home.

Auditory stimulation such as binaural beat (BB) is cost-effective and easy to use (Parodi et al., 2021; Tani et al., 2022). BB stimulation is provided through earphones with slightly different frequencies presented separately to each ear. The brain perceives a sound with a frequency corresponding to the difference between these two frequencies. Its origin is subcortical, specifically in the pons, within the medial nucleus of the superior olivary complex (Sadeghijam et al., 2023). BB stimulation has been shown to modulate brain activity and improve cognitive functions such as working memory and attention (Beauchene et al., 2017; Mujib et al., 2021). Several studies have explored the effects of binaural beats (BB) stimulation on memory and cognitive function in both healthy and clinical populations. Research suggests that BB stimulation enhances episodic and working memory by modulating neural oscillations (Jirakittayakorn and Wongsawat, 2017; Reedijk et al., 2015). For instance, Jirakittayakorn and Wongsawat (2017) demonstrated that BB increased theta power in EEG recordings, which correlated with improved working memory performance. Similarly, alpha-frequency BB has been associated with increased alpha power, promoting attentional focus and relaxation, which may be beneficial for AD patients (Crespo et al., 2013; Park et al., 2018). Reedijk et al. found that alpha-frequency BB improved sustained attention and task performance, likely by enhancing alpha synchronization (Gao et al., 2014; Reedijk et al., 2015). Research also indicates that BB may play a role in cognitive enhancement by promoting neural synchrony (Park et al., 2018). Garcia-Argibay et al. (2019) suggested that BB stimulation could enhance cognitive flexibility and executive function.

However, some studies report no significant cognitive improvements following BB exposure, attributing the discrepancies to variations in individual variability, stimulation frequency, and differences in study design, including variations in EEG recording protocols, cognitive assessment tools, stimulation duration, and control conditions (Chaieb et al., 2015; Vernon et al., 2014). For instance, Goodin et al. (2019) reported no significant effects of BB stimulation on cognitive function, highlighting the need for standardized methodologies in future research. Despite limited research, some preliminary studies suggest that binaural beats BB may influence EEG-derived biomarkers of cognitive decline, such as changes in theta and alpha power, in Alzheimer’s disease (AD) patients. However, the long-term effects and underlying mechanisms of BB in AD remain underexplored (Garcia-Argibay et al., 2019; McMurray, 2006). By employing strict inclusion criteria to ensure participant homogeneity, we hypothesized that BB-induced neurological changes, as measured through EEG analysis, would correlate with significant improvements in cognitive performance in AD patients.

## 2 Materials and methods

The study was conducted at two locations: Dar-ul-Sakoon and Gills Shelter Center. The consent was achieved before the trial from both the administrations of the two facilities. Dar-ul-Sakoon and Gills Shelter Center directed all the related information to their in-charge officials and they were able to assess before and after the experiment which was in line with our ethics guidelines. This approach proactively sought to build on a foundation of collaboration, integrity, and safety by prioritizing the stakeholders’ welfare and success during the research process. The research was approved by the “Research Ethics Committee” at NED University of Engineering & Technology.

### 2.1 Participants

A total of 107 Alzheimer’s disease (AD) patients at Dar-ul-Sukun, Karachi, Pakistan and Gill Shelter Center, Karachi, Pakistan were initially screened for potential inclusion in the study. The experimental procedures were thoroughly explained to all patients by the administration at both centers, along with our research team. The inclusion criteria for the study included a diagnosis of Alzheimer’s disease based on the National Institute of Neurological and Communicative Disorders and Stroke–Alzheimer’s Disease and Related Disorders Association (NINCDS-ADRDA) criteria and the Diagnostic and Statistical Manual of Mental Disorders, Fourth Edition DSM-IV, age 60 years or older, a history of Alzheimer’s disease for at least 5 years, Mini-Mental State Examination (MMSE) scores between 10 and 24 or Clinical Dementia Rating (CDR) scores between 1 and 2, and the ability to provide informed consent, or consent obtained from a legal guardian if the participant was unable.

Exclusion criteria included a diagnosis of other neurological or psychiatric disorders, severe auditory impairments that could interfere with participation in auditory stimulation, the use of medications or therapies that could significantly affect cognitive function, such as nootropic drugs, a history of epilepsy or other seizure disorders, and a lack of interest or refusal to participate in the study.

**FIGURE 1:**
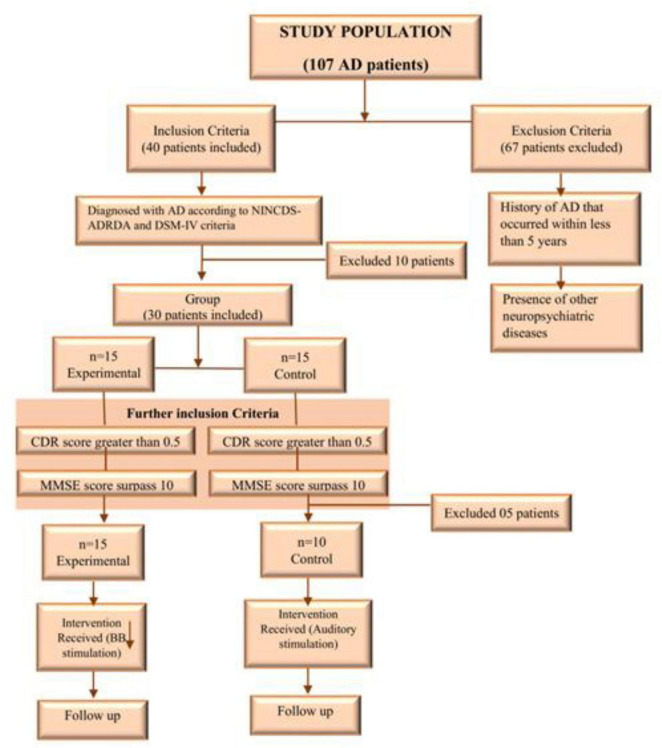
Participant selection criteria for the study.

The participant selection criteria for the study are shown in Figure 1. Of the initial 107 patients, 67 were excluded based on the above criteria, including a lack of interest in participating, having an AD history of less than five years, or the presence of other neurological disorders. The remaining patients were then evaluated using the NINCDS-ADRDA criteria (McKhann et al., 1984) and the DSM-IV (American Psychiatric Association, 1994). Following this evaluation, 30 patients met the inclusion criteria; however, 5 of these were further excluded based on their Mini-Mental State Examination (MMSE) or Clinical Dementia Rating (CDR) scores. Ultimately, 25 patients were selected to participate in the study.

Participants were randomly allocated to the experimental and control groups prior to administering the MMSE for unbiased distribution. The MMSE was then conducted to assess baseline cognitive function. The inclusion/exclusion criteria were pre-defined, as given in Figure 1, to ensure that all participants met the cognitive baseline required for meaningful comparisons and validity of results. A modified minimization approach was employed to allocate participants to either the Experimental or Control group.

The study’s sample size was determined using an interim analysis conducted on the first six participants in each group (12 total), resulting in high effect size (*d* = 1.72) and statistical power (SP = 90%) for significant changes in MMSE scores. The Experimental Group exhibited significant improvements in MMSE scores (Pre-MMSE mean score = 11.57 ± 3.25, Post-MMSE mean score = 17.10 ± 3.18), highlighting the effectiveness of the intervention. Based on these promising results, a maximum sample size of 11 participants per group (total of 22) was calculated to achieve *p* < 0.025 and SP = 80%, ensuring a balance between precision and feasibility.

The interim analysis approach allowed for adaptive refinement of the sample size, optimizing resource utilization while maintaining high statistical rigor. By employing a stricter significance threshold of *p* < 0.025 instead of the conventional *p* < 0.05, the robustness and reliability of the findings were further enhanced, effectively mitigating the potential for false positives. Five participants from the control group did not meet the minimum MMSE qualification criteria necessary for inclusion in the study and were thus excluded thereafter. Despite these exclusions resulting in slightly unequal sample sizes, the study design maintained its robustness due to the randomized allocation process and high statistical power for the sample size. The integrity of the analysis was preserved by adhering strictly to the inclusion and exclusion criteria, ensuring that the findings are both valid and generalizable.

The included 25 AD patients were randomly assigned to either the Experimental-Group (*n* = 15) or the Control-Group (*n* = 10). The Experimental-Group was provided alpha BB stimulation of 10 Hz difference (Left ear: 400 Hz and Right ear: 410 Hz) whereas Control-Group received a created tone comprising the frequency of 400 Hz in both left and right ears. Although the researchers were aware of the group assignments, the administration and the participants in both centers were blinded to their allocation to prevent any bias in their responses.

There were 16 males and 9 females, with an average age of 69.96 ± 8.67 years and an average history of AD of 6.36 ± 1.35 years. The males had a mean age of 71.19 ± 8.28 years and an average history of AD of 6.34 ± 1.52 years, while the female AD patients had mean age of 67.78 ± 9.40 years and an average history of AD of 6.39 ± 1.05 years.

### 2.2 Experimental procedure

**FIGURE 2:**
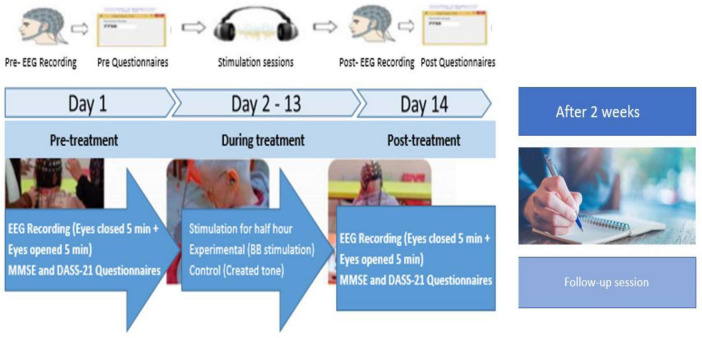
Experiment protocol for experimental- and control groups of AD patients. Pre-Treatment represents psychometric assessments and neurological recording on day 1, During treatment represents BB stimulation days from day 2 to 13 and Post-Treatment represents psychometric assessments and neurological recording on day 14. A follow-up session was taken after two weeks of Post-Treatment, focusing solely on psychometric assessments (MMSE scores).

As shown in Figure 2, the experimental procedure involved a 12-day treatment period with assessments conducted at three key stages: Pre-Treatment (Day 1), Post-Treatment (Day 14), and follow-up session. The Pre-Treatment assessment was carried out one day prior to the start of the treatment, while the Post-Treatment assessment took place one day after the treatment concluded. The follow-up assessment was conducted two weeks later to evaluate the patients’ cognitive progress using the MMSE.

During the treatment period (from Day 2 to Day 13), both groups underwent half-hour daily stimulation sessions. At the Pre-Treatment (Day 1) and Post-Treatment (Day 14) phases, both neurological assessments (EEG recordings) and psychometric assessments (MMSE and Depression Anxiety Stress Scales-21 [DASS-21]) (Al-Shargie and Goh, 2019; Coker et al., 2018; Insel et al., 2006; Pillai et al., 2014; Shigemori et al., 2010; Tierney et al., 2000) were performed. The follow-up session focused solely on MMSE scores. Additionally, participants were asked general questions about their health, daily routine, sleep schedule, activities, and diet to conduct a thorough behavioral and psychometric analysis. These assessments were designed to establish a baseline for each participant’s cognitive and emotional state at the study’s outset.

### 2.3 Implementation steps for intervention

Some challenges were encountered when preparing and attending to most elderly patients, particularly their resistance and anxiety toward the experimental procedures. To address this, we made multiple efforts to build trust and provide reassurance before beginning the experiment. We prioritized clear communication, taking time to thoroughly explain the procedures and address any concerns to ensure the patients felt comfortable and ready to participate. This approach was crucial in upholding ethical considerations and ensuring the patient’s willingness to engage in the study. Throughout the experiment, participants adhered strictly to the protocol under the supervision of qualified neurologists, psychologists, and the nursing staff.

During the test, each participant’s hearing threshold was assessed in a soundproof room using calibrated stereo headphones. The procedure involved presenting pure tones (400 Hz and 410 Hz) at low-intensity levels, starting below the expected threshold (e.g., 0–10 dB), and gradually increasing the intensity in 5 dB increments until the participant could reliably detect the sound. The lowest intensity level at which the participant could detect the tone 50% of the time was recorded as their hearing threshold for that frequency.

The device threshold was calibrated to present the stimuli at a level 50 dB above the hearing threshold. For instance, if the given criteria for 400 Hz for a particular subject was 15 dB, the sound was produced at 65 dB (15+50). Subsequent changes were made to both amplification levels based on participant input to maintain the volume at a loud but comfortable level. This process ensures that the auditory stimuli were both detectable and comfortably loud, tailored to each participant’s auditory sensitivity. This auditory test was conducted on Day 1.

### 2.4 Generation of BB/standard auditory tone stimulation

BB is created by presenting two slightly different-frequency sound waves to each ear separately. The brain then perceives a third sound, which is the difference between the two frequencies. For example, if one ear hears a 400 Hz tone and the other ear hears a 410 Hz tone, the brain perceives a beat at a frequency of 10 Hz (Mujib et al., 2021; Sadeghijam et al., 2023). Both types of stimulation tones (BB/Standard auditory tone stimulation) were created using Adobe Audition v3.0 (Tierney et al., 2000). The stimuli were presented at a minimum intensity of 50 dB above each participant’s hearing threshold, determined through standardized pure-tone audiometry (Goodin et al., 2019).

### 2.5 Assessments

The study utilized MMSE and DASS-21 questionnaires to assess the cognitive and psychometric effects of BB and standard auditory stimulation, respectively. The MMSE is a widely used screening tool for assessing cognitive characteristics (Al-Shargie and Goh, 2019; American Psychiatric Association, 1994; Shigemori et al., 2010) and assessing areas of cognitive functioning including orientation, language, attention, calculation, registration, recall, and copying (Pillai et al., 2014; Shigemori et al., 2010; Tierney et al., 2000). Each task is scored based on the individual’s performance, with a maximum score of 30 points indicating normal cognitive function. The MMSE questions were asked orally and the particular questions involving writing, reading, and drawing were carried out on paper, with the total duration for each patient ranging from 30 to 40 min. MMSE is categorized into different levels of cognitive impairment. The scores 21–26 represent mild impairment, 10–20 indicate moderate impairment, 10–14 suggest moderately severe impairment, and scores below 10 signify severe impairment (Al-Shargie and Goh, 2019; Pillai et al., 2014; Shigemori et al., 2010; Tierney et al., 2000). The DASS-21 is a 21-item questionnaire that measures symptoms associated with depression, anxiety, and stress (Insel et al., 2006). The predictive validity of the DASS-21 has also been studied in AD patients (Tierney et al., 2000). This test was conducted verbally and lasted approximately half an hour. The degree of application of each statement is indicated by a rating on a scale from 0 to 3. Scores range from 0 for “not applied” to 3 for “sometimes applied” (Coker et al., 2018; Insel et al., 2006; Pillai et al., 2014).

### 2.6 Binaural beat/auditory device usability

To explore the usability of the Binaural Beat delivered through stereo headsets, participants were given the System Usability Scale (SUS) questionnaire at the end of the evaluation (Marijanović et al., 2021). The SUS itself, consisting of 10 items with Likert scale responses ranging from 1 (strongly disagree) to 5 (strongly agree), was implemented. If the SUS score is 66 there is no reason to worry because it indicates good usability. To SUS (system usability scale) rating’s calculation is done by the sum of the odd-numbered questions rating and then five is subtracted from it. Then the sum of the even-numbered questions rating minus twenty-five is taken to find out the SUS rating. In addition, the total of the first and last SUS ratings from both odd and even-numbered questions is multiplied by 2.5 to discover the final SUS composite rating (Eq. 1) (Brooke, 1996; Mujib et al., 2023).


(1) SUSRating=[(SumofoddQuestions-5)+

(25-SumofevenQuestions)]*2.5

### 2.7 EEG recording

To investigate brain activity, this study used an EEG Mitsar-NVX from 38 scalp locations (FP1, FPZ, FP2, F7, F3, FZ, F4, F8, FT7, FC3, FCZ, FC4, FT8, T3, TP7, T5, T4, TP8, T6, C3, CZ, C4, CP3, CPZ, CP4, P3, PZ, P4, P5, PO3, POZ, PO4, P6, PO7, PO8, O1, OZ, O2) with two reference electrodes (A1, A2) following the standardized 10–20 international system (Rao and Hasan, 2021). EEG recordings were obtained during phases on day 1 and 14. The sampling frequency is set at 500 Hz. The saline liquid is used as an electrolytic gel to obtain good conductivity. Data is acquired in a quiet and ventilated room to minimize noise.

### 2.8 EEG signal processing

Raw EEG data was subjected to detrending to remove a DC offset and thereby adjust the baseline. A 5th-order Butterworth band-pass IIR filter (1–45 Hz) was used to eliminate the 50 Hz artifact. The sampling frequency is set at 500 Hz. By adjusting the filter order *n*, frequency range Xa, and filter type ftype, the MATLAB command “butter (a, Xa, ftype)” was used to calculate the filter coefficients. The required filtered output was then obtained using the command “filter (b, c, x),” where b and c are filter coefficients and x is raw EEG data as input. Based on a visual inspection, EEG data with eye blinks, ocular movements, and EMG artifacts were eliminated. The power spectral density of EEG signals was determined by utilizing Welch’s method carried out in Matlab. To enhance estimation quality by controlling spectral leakage and data variance sliding Hanning window of 4 s, with a cross-over of 2 s, was applied. The relative EEG power was computed by normalizing the absolute power within each frequency band (θ: 4–8 Hz, α: 8–12 Hz, β: 13–30 Hz, and γ:30–45 Hz) using the total absolute power ranging across all channels within 2 to 45 Hz.

### 2.9 Statistical analysis

The demographic and baseline performance data of all 25 participants which include participants’ age, MMSE score, and DASS-21 score in Pre-Treatment (Day-1) were compared between both groups (Experimental- and Control-Group) using an unpaired *t*-test (*p* < 0.05). The Wilcoxon signed-rank test was applied to compare changes in MMSE score and DASS-21 score between Post-Treatment (Day 14) and Pre-Treatment (Day 1) for both groups (Experimental- and Control-Group). A two-way ANOVA was conducted to examine the effects of groups (Experimental- and Control-Group) and Sessions (Pre-Treatment, Post-Treatment, and follow-up session) on the MMSE score. The student *t*-test was applied to SUS scores of Post-Treatment against the acceptable usability level of 66 for both groups (experimental- and control groups). However, a paired *t*-test was applied to compare the relative EEG power recorded in Pre-Treatment (Day 1) with the power recorded in Post-Treatment (Day 14) in both eyes opened and eyes closed states.

The Cohens method was applied to find the effect size and to demonstrate whether the effects of training have practical importance; ensuring that significant behavioral and neurological changes in Post-Treatment (Day 14) are not due to false positives (Homan, 1988). The effect size was calculated for changes in MMSE scores and EEG relative power. The mean of the two groups were subtracted and divided with a pooled standard deviation [see Eq. (2)]. The values of the effect size larger than 0.8 were considered as large while values between 0.4 and 0.8, and less than 0.4 were considered as medium and low effect sizes.


(2) $$c\,o\,h\,e\,n\,d=\frac{{\bar{X}}_{1}-{\bar{X}}_{2}}{\sqrt{\frac{S\,D_{1}\,\left(n_{1}-1\right)+S\,D_{2}\,\left(n_{2}-1\right)}{n_{1}+n_{2}-2}}}$$

Where $\bar{\text{x}}$ are mean values, {SD}_1,2_ are standard deviations, and n_1,2_ are sample sizes of two variables.

Statistical Pearson correlation tests were computed to examine the correlation between MMSE Components (orientation, language, attention, calculation, registration, recall, and copying) and PSD of each EEG channel for four frequency (θ, α, β, and γ) bands using Eq. 3, for both Experimental- and Control-Groups. Statistical significance was set at *p* < 0.05 for all tests, and multiple comparisons were corrected to control the expected proportion of false positives using the False Discovery Rate (FDR) method. All the statistical analyses were carried out in MATLAB software.


(3) $$r=\frac{\sum \left(X\,i-X^{\prime }\right)\,\left(Y\,i-Y^{\prime }\right)}{\sqrt{\sum {\left(X\,i-X^{\prime }\right)}^{2}\,{\left(Y\,i-Y^{\prime }\right)}^{2}}}$$

Where, *r* represents the correlation coefficient, Xi and Yi are individual data points, and X‘ and Y‘ are means of X and Y, respectively.

## 3 Results

The baseline demographic data (age, MMSE, and DASS-21 scores) of both the Experimental and Control groups were compared using an unpaired *t*-test (*p* < 0.05) to confirm no significant differences at the start of the study. This ensures that any observed effects can be attributed to the treatment rather than pre-existing group differences.

Table 1 shows a comprehensive analysis of the demographic characteristics of the AD patients’ cohort, including age, gender distribution, and duration of AD. Table 2 presents the MMSE scores for participants in both the experimental group (patients # 1 to 15) and Control-Group (patients # 16 to 25) across three time points; Pre-Treatment, Post-Treatment, and follow-up sessions. Patients # 26 to 30 were assessed but did not qualify to receive further interventions (stimulation) due to their low baseline scores, which excluded them from subsequent sessions.

**TABLE 1:** Demographic characteristics of the AD patients.

Alzheimer’s patients				
Patient numbers and age (years)		25 (69.96 ± 8.67 years)		
		**Male**	**Female**	
		16 (71.19 ± 8.28 years)	9 (67.78 ± 9.40 years)	
Duration of disease—year (SD)		6.36 ± 1.35 years		
		**Male**	**Female**	
		6.34 ± 1.52 years	6.39 ± 1.05 years	
Education—year (SD)		13.6 ± 4.2 years		
DASS-21		**Stress**	**Anxiety**	**Depression**
	Total	10.44 ± 1.2	9.64 ± 0.9	12.96 ± 1.14
	Experiment group	10.51 ± 1.4	9.57 ± 0.92	13.04 ± 1.14
	Control group	10.39 ± 1.37	9.67 ± 0.85	12.92 ± 1.14
MMSE	Total	14.02 ± 3.16		
	Experiment group	13.94 ± 5.6		
	Control group	14.1 ± 4.2		
CDS Level		**1**	**2**	
		*n* = 21	*n* = 4	

**TABLE 2:** Change in MMSE scores across sessions for AD patients, showing Pre-Treatment, Post-Treatment, and follow-up.

Experimental-group					Control-group		
**Patient #**	**Pre-Treatment**	**Post-Treatment**	**Follow-up session**	**Patient #**	**Pre-Treatment**	**Post-Treatment**	**Follow-up session**
1	11	19	14	16	16	18	16
2	11	15	13	17	15	16	16
3	19	22	20	18	20	17	19
4	11	16	12	19	11	13	9
5	14	18	16	20	11	12	13
6	11	13	12	21	16	12	13
7	14	16	18	22	17	16	18
8	19	22	20	23	18	21	17
9	19	21	20	24	11	12	12
10	17	19	19	25	11	14	12
11	15	18	16	*26	9	Excluded due to less MMSE (< 10) score	
12	11	20	15	*27	9		
13	13	19	14	*28	8		
14	15	22	16	*29	9		
15	17	22	19	*30	7		

The patient numbers (#) are given while the ‘*’ indicates the patients who were excluded due to having lower MMSE (<10) score.

### 3.1 Psychological assessments

#### 3.1.1 MMSE

A two-way ANOVA (as shown in Table 3) was conducted to examine the effects of groups (Experimental- and Control-Group) and Sessions (Pre-Treatment, Post-Treatment, and follow-up session) on the MMSE score. The analysis revealed a statistically significant main effect of the group, *F* = 6.08, *p* = 0.016, with a partial eta-squared (h^2^) of 0.063 indicating that 6.3% of the variance in the MMSE score can be attributed to differences between Experimental and Control-Group. Similarly, there was a significant main effect of session, *F* = 3.859, *p* = 0.024, with partial eta-squared (h^2^) of 0.079, accounting for 7.9% of the variance. However, the interaction between the group and the session was not significant.

**TABLE 3:** Two-way ANOVA on MMSE score with F-statistic, *p*-value, and effect size (h^2^).

Factors	F	*p*-value	h^2^
Groups (experimental- and control-group)	6.087	0.0161	0.063
Session (Pre-Treatment, Post-Treatment, follow-up session)	3.859	0.0249	0.078
Group*session	2.358	0.102	0.049

**FIGURE 3:**
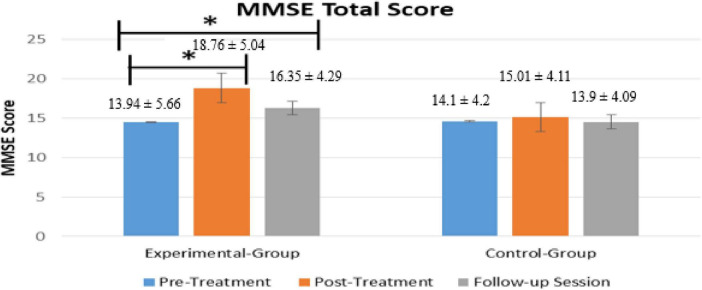
Comparison of MMSE total within group scores between Pre- and Post-Treatment phases. Bars represent the mean and SD of Pre-Treatment score (blue bars), Post-Treatment score (orange bar), and Follow-up score (gray bar). * Indicate the significant difference (*p* < 0.025) in MMSE score between the Post-Treatment (Day 14) and follow-up sessions as compared to Pre-Treatment (Day 1) in both groups.

Figure 3 displays the total score of MMSE within the group (for both Experimental- and Control-Group). In the Experimental-Group, the Post-Treatment MMSE score significantly increased (*p* = 0.00000012, *t*-value = 7.33) as compared to the Pre-Treatment MMSE score while no significant improvements were noted in the Control-Group (*p* = 0.2306). Similarly, Follow-up MMSE scores in the experimental group (*p* = 0.00000077, *t* = 7.27) significantly increased compared to Pre-Treatment scores, with no significant improvement observed in the control group (*p* = 0.4236).

**FIGURE 4:**
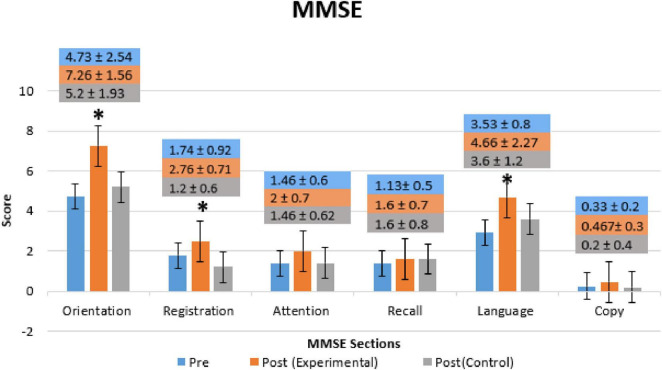
Mini-Mental State Exam (MMSE) mean scores of each section (Orientation, Registration, Recall, Attention, Language, and Copying) for Pre- and Post-Treatment (Day 14) of Experimental- and Control-Group. Bars represent the mean and SD of Pre-Treatment (blue bars), Post-Treatment of the Experimental-Group (orange bars), and Post-Treatment of Control-Group (gray bars). * Indicate the significant difference (*p* < 0.025) in MMSE score between the Post-Treatment (Day 14) as compared to Pre-Treatment (Day 1) in both groups.

Figure 4 shows a comparison of MMSE mean scores of both groups (Experimental- and Control groups) with the Pre-Treatment session for all MMSE sections (Orientation, Registration, Recall, Attention, Language, and Copying). In the experimental group, significant improvement was observed in the patients’ orientation (*p* = 0.00015, *t*-value = 1.687), registration (*p* = 0.0213, *t*-value = 1.688), and language (*p* = 0.0182, *t*-value = 1.703). However, other parts of the MMSE, such as attention, recall, and copy, did not reveal significant improvement after the BB session. In the control group, a significant decline in activity was observed only in the patients’ registration (*p* = 0.04122, *t*-value = −1.705) but, no significant changes were observed in other sections.

#### 3.1.2 DASS-21

**FIGURE 5:**
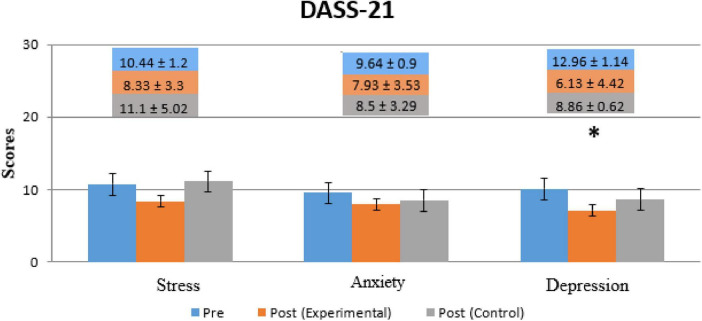
Comparison of patients’ scores for the DASS-21 questionnaire for all three conditions (Depression, Anxiety, and Stress). The Blue Bar represents Pre-Treatment, the orange bar represents Post-Treatment (Experimental group) and the Gray bar represents Post-Treatment (Control group). * Indicates the significant difference (*p* < 0.05) in DAAS score between Post-Treatment (Day 14) as compared to Pre-Treatment (Day 1) in both groups.

Figure 5 shows the comparison of DASS-21 within (for both the experimental and control groups) in both Pre- and Post-Treatment for all subparts of DASS-21’s score (Depression, Anxiety, and stress). Depression significantly decreased in the experimental group (*p* = 0.0235, *t* = 1.701) while no significant improvements were observed in the control group or the other subparts of both groups. A significant level was set at *p* < 0.05.

### 3.2 EEG power spectrum

**FIGURE 6:**
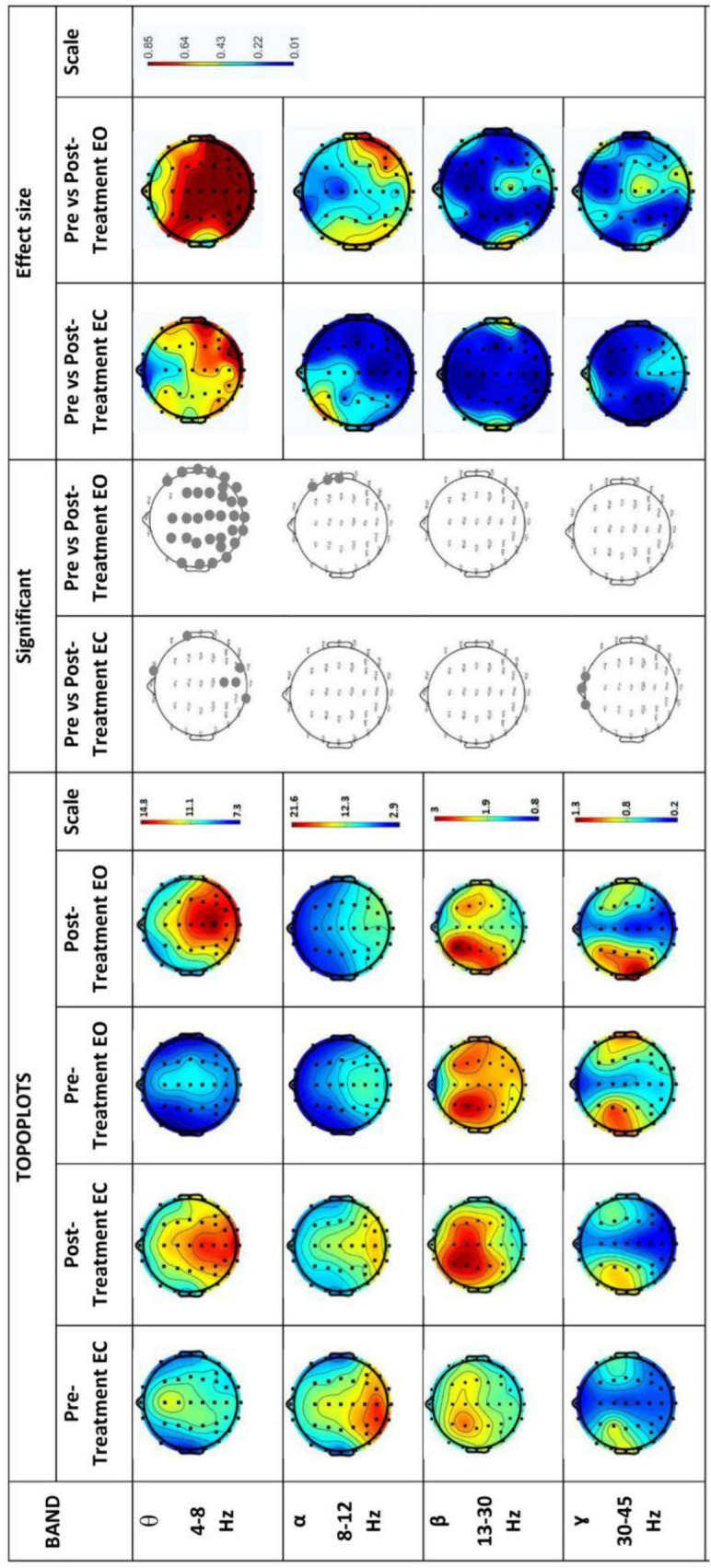
EEG power spectrums for Experimental-Group and statistical comparisons between states in four frequency bands (θ, α, β, and γ). Rows represent frequency bands (first row: θ-band, second row: α-band, third row: β-band, fourth row: 7-band). Columns represent the power spectrum in four states (Pre-Treatment EC; first column, Post-Treatment EC; second column, Pre-Treatment EO; third column and fourth column; Post-Treatment EO), statistical comparisons between Pre-Treatment EC vs. Post-Treatment EC and Pre-Treatment EO vs. Post-Treatment EO states (significant changes in column 5 and 6, respectively) and effect size between Pre-Treatment EC vs. Post-Treatment EC and Pre-Treatment EO vs. Post-Treatment EO states (column 7 and 8, respectively). Gray dots represent a significant increase and black dots represent a significant decrease.

Figure 6 shows EEG power spectrums for the experimental group in both eyes opened (EO) and eyes closed (EC). Rows represent frequency bands (first row: θ-band, second row: α-band, third row: β-band and fourth row: 7- band). θ-band power increased significantly (*p* < 0.05) with high effect size in the temporal, parietal, and occipital region in the Post-Treatment EO BB stimulation session which significantly increased only in the occipital region in the Post-Treatment EC BB stimulation session. A decrease in power was detected in the α-band during the Post-Treatment EO BB state compared to the Pre-Treatment EO BB state specifically in the temporal region. Conversely, an increase in power was observed in the γ-band, primarily localized to the frontal regions in the Post-Treatment EC BB stimulation session. No changes were observed in β-band in both Pre- and Post-Treatment BB stimulation.

**FIGURE 7:**
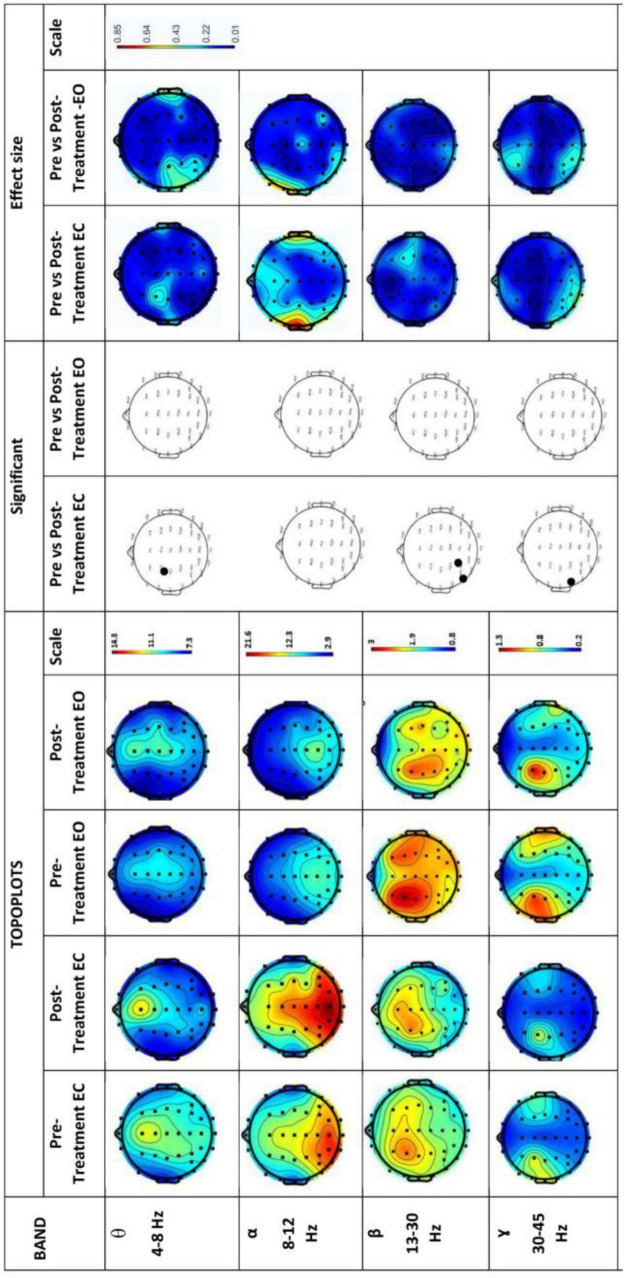
EEG power spectrums for Control group and statistical comparisons between states in four frequency bands (θ, α, β, and γ). Rows represent frequency bands (first row: θ-band, second row: α-band, third row: β-band, fourth row: 7-band). Columns represent the power spectrum in four states (Pre-Treatment EC; first column, Post-Treatment EC; second column, Pre-Treatment EO; third column and fourth column; Post-Treatment EO), statistical comparisons between Pre-Treatment EC vs. Post-Treatment EC and Pre-Treatment EO vs. Post-Treatment EO states (significant changes in column 5 and 6, respectively) and effect size between Pre-Treatment EC vs. Post-Treatment EC and Pre-Treatment EO vs. Post-Treatment EO states (column 7 and 8, respectively). Gray dots represent a significant increase and black dots represent a significant decrease.

Figure 7 shows EEG power spectrums for the control group. Rows represent frequency bands (first row: θ-band, second row: α-band, third row: β-band and fourth row: 7- band). In contrast to the experimental group, the neurological findings in the control group indicated no observable changes in all bands.

### 3.3 Correlation between MMSE and PSD of EEG channels

**FIGURE 8:**
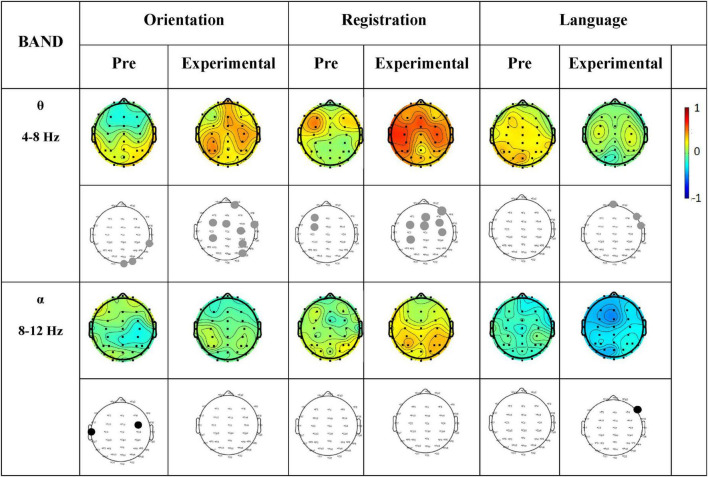
Correlation between MMSE scores (Orientation, Registration, and Language) and PSD of each EEG channel for Pre-Treatment and Post-Treatment (Experimental group). Columns represent the sessions (Pre-Treatment and Post-Treatment experimental group) of MMSE score (Orientation, Registration, and Language) of frequency bands (θ and α). The first row in each frequency band represents the correlation values, while the second row shows the significance values. Gray dots indicate a significant positive correlation, and black dots indicate a significant negative correlation. The scale from –1 to 0 represents a negative correlation, while the scale from 0 to 1 represents a positive correlation.

A correlation analysis was subsequently performed between the MMSE subtest scores and the EEG θ-band and α-band activity. Figure 8 shows the correlation between the subtest scores on MMSE and activity in specific frequency bands of the Pre-Treatment EO state, and Post-Treatment EO states of the experimental group.

We found a positive correlation (significantly *p* < 0.05) in θ-band in the frontal region of the brain during language tasks and in the frontal and central regions during registration and orientation tasks. In addition, no correlation was observed in the α- band.

### 3.4 Usability testing

The SUS scores information shows that 86% of AD patients had positive feedback which was above the threshold of 66 which is the acceptable value. Besides, a significant difference in SUS average scores was determined in both the experimental- and control groups, which were widely beyond the acceptable usability level. Both the experimental (*p* = 0.024, *t*-value = 2.88) and the control (*p* = 0.023, *t*-value = 2.667) groups produced significant differences.

## 4 Discussion

This study aimed to investigate the effects of BB stimulation on enhancing cognitive functions of AD patients. BB stimulations corresponding to θ and α- brain waves have shown a beneficial influence on cognitive processes (Cohen, 1988; Cruceanu and Alpha, 2013; Wianda and Ross, 2019). Previous studies have recommended BB stimulation at a frequency of 10 Hz yielded a positive effect on the working memory capacity. Consequently, in this study, the AD patients in the Experimental-Group received 10 Hz BB stimulation. We found improved cognitive and psychometric scores followed by increased θ band activity and a modified correlation between MMSE scores and global θ-band activity.

The observed behavioral outcomes in the experimental group which underwent BB stimulation demonstrated significant improvements. The substantial decrease in depression and stress scores, as indicated by the DASS-21 scale, aligns with research suggesting that auditory interventions can positively impact emotional wellbeing (Le Scouarnec et al., 2001; Puzi et al., 2013; Sung et al., 2017). These findings highlight the potential of BB stimulation to reduce emotional distress in AD patients experiencing severe mood disturbances as part of their dementia-related symptoms (Babulal et al., 2016).

Furthermore, behavioral changes were also observed in the experimental group by MMSE indicating a meaningful impact of the BB stimulation. The consequences of AD include cognitive decline, behavioral change, memory loss, and communication. The improved score in components of the MMSE examination following intervention demonstrates that BB has the potential to facilitate advancements in orientation, registration and language. These findings align with studies conducted on non-AD patients which explore the cognitive benefits of BB interventions in dementia-related disorders (Beauchene et al., 2016; Beauchene et al., 2017; Kraus and Porubanová, 2015). However, there were no significant impacts in the control group either in depression, anxiety, and stress levels or in any part of MMSE.

The EEG recordings in the experimental group provide insight into the underlying neurological mechanisms contributing to the observed behavioral improvements. The significant increase in θ- band power in the occipital region of the brain coincides with research (Sarnthein et al., 1998; Sauseng et al., 2010; Zhang et al., 2016) suggested showing θ- oscillations have been connected to cognitive functions like attention, and memory. θ- activity has been reported to play a major role in working memory functions (Gevins and Smith, 2000; Kraus and Porubanová, 2015; Zhang et al., 2016) and in the integration of different neural circuits during memory processes (Kraus and Porubanová, 2015). The results of previous research (Sammer et al., 2007) have shown that an increase in the synchronization of oscillatory phases between different brain regions supports working memory and acts by facilitating neural connections. Phase synchronization in the θ- frequency range persists between the prefrontal and parietal brain regions throughout the stages of a working memory task, including encoding, maintenance, and retrieval (Fell and Axmacher, 2011).

The observed decrease in α-band power, despite the use of α-frequency BB stimulation may indicate that the brain is reallocating cognitive resources to more demanding tasks. A reduction in α-band power is often associated with increased cognitive engagement, suggesting the brain enters a more active state that facilitates processes such as attention and memory (Beauchene et al., 2016; Cruceanu and Alpha, 2013; McKhann et al., 1984; Sauseng et al., 2004). This shift in neural activity could account for the simultaneous increase in θ-band power, which is linked to memory and attentional processes, reflecting an adaptive response to BB stimulation aimed at enhancing cognitive function (Chaieb et al., 2015; Garcia-Argibay et al., 2019).

The results of this study align with those stated by Christopher (Desai et al., 2015), providing further support in the role of θ-band activity modulation in enhancing cognitive functions through binaural beats stimulation. While our study did not observe significant evidence of improvement in attention, as noted in prior studies, we found significant changes in orientation, registration, and language in the Experimental Group, suggesting that θ-band activity modulation contributes to specific cognitive improvements. Our findings follow the path taken by the abovementioned variation wherein the frontal and temporal regions show an increase in correlation score after the BB stimulation. Moreover, in AD patients, BB stimulation appeared to have a positive effect on neural network efficiency with θ-band correlation increasing during cognitive tasks, which was particularly prominent in brain regions related to learning and memory (Benwell et al., 2020; Klimesch, 1999). The prevalence of left hemisphere activity in the θ-band during cognitive tasks underlines the high importance of BB stimulation on speech, meaning processing, and concentration in AD subjects (Fonseca et al., 2011; Gianotti et al., 2007; Karakaş et al., 2003; Nokia et al., 2012).

The findings of this study hold significant implications for diagnosing AD and identifying its biomarkers. By analyzing MMSE scores, brainwave activity (including changes in α- and θ-band power), and cognitive performance across various tasks, the study uncovers specific EEG patterns linked to cognitive decline in AD patients. These neurophysiological markers can help distinguish AD patients from healthy controls. By demonstrating the potential of BB to improve cognitive function, as shown by enhanced MMSE scores, this research provides valuable insights into non-invasive treatment options for AD patients. Moreover, the methods used to implement BB stimulation in this study offer a practical framework that can be adopted by other clinics to incorporate BB into patient care protocols, potentially improving treatment outcomes.

This study was a single-blind pilot/feasibility investigation, and as such, effect sizes may be smaller in larger, more rigorously controlled studies. Therefore, the findings should be regarded as preliminary and as a foundation for future research. To enhance scientific rigor and expand understanding, future studies could explore the combination of therapies such as tDCS (Brown, 2007), transcutaneous electrical nerve stimulation (Abul Hasan et al., 2023; Mujib et al., 2024), and neurofeedback (Zafar et al., 2025), along with the integration of machine learning approaches (Ather et al., 2024; Marappan et al., 2022; Zahid Rao et al., 2024).

In conclusion, this research investigated the BB effect on cognitive scores, particularly attention and working memory, in patients with AD. Further research is warranted to elucidate its therapeutic mechanisms comprehensively and explore its applicability.

## Data Availability

The datasets generated and/or analyzed during the current study are not publicly available due to privacy and ethical concerns but are available from the corresponding author on reasonable request. Requests to access the datasets should be directed to abulhasan@neduet.edu.pk.
